# Experimental setup for high-temperature *in situ* studies of crystallization of thin films with atmosphere control

**DOI:** 10.1107/S1600577520010140

**Published:** 2020-08-21

**Authors:** Anders Bank Blichfeld, Kristine Bakken, Dmitry Chernyshov, Julia Glaum, Tor Grande, Mari-Ann Einarsrud

**Affiliations:** a Department of Materials Science and Engineering, NTNU Norwegian University of Science and Technology, Sem Saelands vei 12, Trondheim 7491, Norway; b Swiss–Norwegian Beamlines, European Synchrotron Radiation Facility, 71 avenue des Martyrs, Grenoble 38043, France

**Keywords:** synchrotron X-ray diffraction, high-temperature thin-film environments, chemical solution deposition, thin films, *in situ* studies

## Abstract

Understanding the crystallization process for chemical solution deposition (CSD) processed thin films is key in designing the fabrication strategy for obtaining high-quality devices. Here, an *in situ* sample environment is presented for studying the crystallization of CSD processed thin films under typical processing parameters using near-grazing-incidence synchrotron X-ray diffraction. Typically, the pyrolysis is performed in a rapid thermal processing (RTP) unit, where high heating rates, high temperatures and atmosphere control are the main control parameters. The presented *in situ* setup can reach heating rates of 20°C s^−1^ and sample surface temperatures of 1000°C, comparable with commercial RTP units. Three examples for lead-free ferroelectric thin films are presented to show the potential of the new experimental set-up: high temperature, for crystallization of highly textured Sr_0.4_Ba_0.6_Nb_2_O_6_ on a SrTiO_3_ (001) substrate, high heating rate, revealing polycrystalline BaTiO_3_, and atmosphere control with 25% CO_2_, for crystallization of BaTiO_3_. The signal is sufficient to study a single deposited layer (≥10 nm for the crystallized film) which then defines the interface between the substrate and thin film for the following layers. A protocol for processing the data is developed to account for a thermal shift of the entire setup, including the sample, to allow extraction of maximum information from the refinement, *e.g.* texture. The simplicity of the sample environment allows for the future development of even more advanced measurements during thin-film processing under non-ambient conditions.

## Abstract 2

A high-temperature setup is presented for *in situ* synchrotron scattering experiments on thin films, including high heating rates and atmosphere control.

## 1. Introduction   

There has been a significant push for creating new *in situ* and *in operando* measurement sample environments for diffraction studies using synchrotron radiation in recent years, as studies under actual synthesis and operating conditions or under other external stimuli are needed for the development of advanced materials, replacing the more traditional static measurements. Such *in situ* and *in operando* studies have given valuable new insight into the synthesis of materials, *e.g.* under hydro- and solvo-thermal conditions (Millange *et al.*, 2010 ▸; Jensen *et al.*, 2012 ▸; Skjaervø *et al.*, 2018 ▸), catalysts under operating conditions (Frenkel *et al.*, 2012 ▸; Andersen *et al.*, 2017 ▸) and materials under high pressure (Jiang *et al.*, 2016 ▸; Briggs *et al.*, 2019 ▸).

When a material is deposited onto a surface, new opportunities emerge for tuning the functionality. The plethora of possible deposition methods makes it difficult to design a single experimental setup that is suitable for more advanced structural studies and they are often limited by the physical requirements of the specific deposition method, *e.g.* a vacuum. At the same time, positioning becomes an issue once the sample is inhomogeneous regarding orientation. A number of examples can be found in the literature, such as physical vapor deposition with sputtering (Matz *et al.*, 2001 ▸; Walter *et al.*, 2015 ▸; Highland *et al.*, 2015 ▸) and diffusion cells (Weber *et al.*, 2009 ▸), and chemical solution deposition (CSD) with pre-spin-coated films for *in situ* annealing (Nittala *et al.*, 2012 ▸, 2013 ▸) and even during solution deposition (Miyadera *et al.*, 2015 ▸).

**Figure 1:**
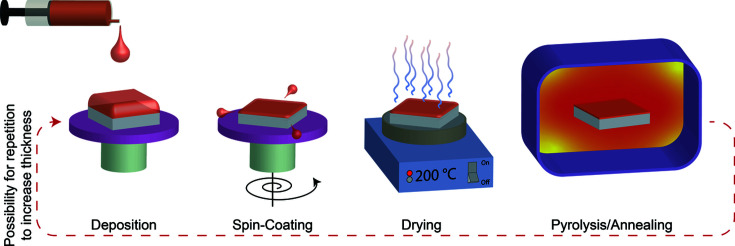
An illustration of the different steps in a typical CSD process. The dashed red arrow indicates the option to repeat the process to increase film thickness.

Functional oxide materials are used as crystalline textured or epitaxial films (<500 nm to several micrometres) on substrates, *e.g.* in electronics. The semiconductor industry has promoted intensive research on thin functional oxide films as CSD (Schwartz *et al.*, 2004 ▸) offers the advantages of simplicity and versatility in the manufacturing process. A solution containing the precursors is deposited onto a substrate. The deposited wet film is dried, annealed further at medium temperature (400–600°C) to decompose the precursors (pyrolysis), and finally crystallized and densified at even higher temperature (>700°C), see Fig. 1 ▸. Several deposition and heating steps are necessary to provide the necessary film thickness.

CSD methods are scalable and cheap processes (Schwartz *et al.*, 2004 ▸; Sunde *et al.*, 2016 ▸), but a challenge with the CSD methods often used in the literature (Bretos & Calzada, 2011 ▸) is the application of toxic/harmful organic solvents not compatible with green chemistry. CSD methods based on aqueous solutions do, on the other hand, provide a green alternative (Bhuiyan *et al.*, 2006 ▸). Generally, a low heat treatment temperature favors heterogeneous crystallization while a higher temperature favors homogeneous crystallization, due to the lower activation energy for heterogeneous crystallization than for homogeneous (Schwartz *et al.*, 2004 ▸).

One way of thermally processing CSD thin films is by using a rapid thermal processing (RTP) unit, also known as rapid thermal annealing furnace. They offer very high heating rates, high temperatures, small thermal masses and atmosphere control, which can be beneficial for fabricating highly textured oxide thin films compared with using a conventional furnace (Bassiri-Gharb *et al.*, 2014 ▸; Queraltó *et al.*, 2016 ▸). Depending on the RTP model, the heat source can be placed at various positions relative to the sample, but the normal configuration is an array of infared light bulbs at a distance above the surface.

Aqueous CSD for preparing lead-free ferroelectric thin films has a high priority in our group and we have developed reliable synthesis protocols for a number of material systems: BaTiO_3_ (Raeder *et al.*, 2018 ▸), K_1−*x*_Na_*x*_NbO_3_ (Gaukås *et al.*, 2019 ▸; Pham *et al.*, 2019 ▸) and Bi_0.5_Na_0.5_NbO_3_ (Christensen *et al.*, 2017 ▸). This has given rise to several unanswered questions. With the large difference in crystallization temperature and melting point for metal oxide CSD thin films, there is a large driving force for the precipitation of small crystallites, but at the same time a large probability of the formation of unwanted metastable secondary phases that characterizes diffusion-limited crystallization (Lange, 1996 ▸). Hence there is a need to study the preparation of films made by CSD using *in situ* techniques to reveal their nucleation and crystallization behavior in order to modify the processing or synthesis to tailor the film properties with respect to epitaxy, crystallinity or orientation.

In this work, we present our newly developed *in situ* cell for studying the crystallization of CSD thin films using synchrotron X-ray diffraction under similar pyrolysis/annealing conditions to an RTP unit, where temperatures >800°C, high heating rates ∼20°C s^−1^ and atmosphere control are the most important parameters. The setup was designed to be suitable to study any type of annealing or heat treatment of thin films under a controlled atmosphere, but in this study ferroelectric films made by CSD are used as examples. Here we present three case studies using the *in situ* rapid heating setup, showing the potential applications and possibilities for the developed approach. One example of a high heating rate and temperature is illustrated with a single layer of an Sr_0.4_Ba_0.6_Nb_2_O_6_ (SBN) thin film deposited on a (100)-oriented SrTiO_3_ (STO) substrate. The second example shows how the transformation of polycrystalline BaTiO_3_ (BTO) to epitaxial can be captured during crystallization. The third example demonstrates the use of a controlled atmosphere during the annealing, here a high concentration of CO_2_, and how that influences the crystallization of BTO.

## 2. Experimental   

For typical CSD processed thin films, the number of depositions is determined by the desired thickness of the film, but here only a single as-deposited layer was used for the studies, to give an insight into the crystallization occurring during the pyrolysis/annealing step of the initial layer. For studying the crystallization of such thin films (∼20 nm after pyrolysis) the flux, the optics and a time resolution of seconds are important, meaning that these types of studies and the data quality can be substantially improved by conducting them at synchrotrons and using a large 2D detector. The experiments were performed at SNBL@ESRF, taking advantage of the PILATUS 2M detector (Dectris) and the flexible infrastructure available on the beamline with respect to adapting the sample area and customizing the data acquisition (Dyadkin *et al.*, 2016 ▸). The PILATUS 2M detector has an active area of 254 mm × 289 mm, with a resolution of 1475 × 1679 pixels, a pixel size of 172 µm × 172 µm comprised of 3 × 8 modules, and a maximum frame rate of 30 Hz (Dectris, 2013 ▸).

### 2.1. The setup   

**Figure 2:**
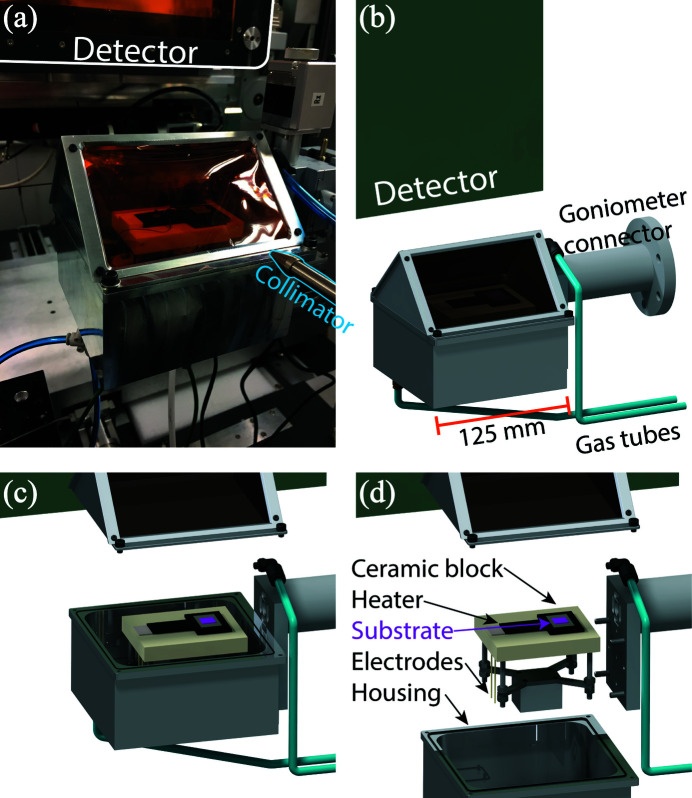
(*a*) A photograph of the setup with the controlled atmosphere option on SNBL@ESRF. (*b*) A schematic drawing with the controlled atmosphere option. (*c*) A view underneath the lid for the controlled atmosphere. (*d*) Further details of the inside of the setup.

Fig. 2 ▸ shows the *in situ* thin film rapid heating setup built around a flat heating element of 850 W from Bach Resistor Ceramics GmbH, with an active area of 25 mm × 30 mm providing heating rates of up to 20°C s^−1^ and a maximum surface temperature of the sample of 1100°C. The surface temperature was measured by a 0.25 mm thick type-K thermocouple that also acted as a spring load to hold the sample in place, and which was therefore in direct contact with the sample. The heating element was placed in a block of Fiberfrax Duraboard as thermal insulation. When using atmosphere control, a machined block of Macor was used as thermal insulation to lower the surface area and porosity for faster exchange of the atmosphere. The insulation block was placed on four threaded steel rods for further isolation from the aluminium box and for height adjustment. The outer dimensions of the surrounding box are 125 mm × 125 mm × 115 mm with a wall thickness of 4 mm. Feedthroughs for gas and electrical wires were placed in the bottom of the box. To allow for a controlled atmosphere, a lid was designed with two large and flat Kapton tape windows for maximal access to reciprocal space [Figs. 2 ▸(*a*) and 2 ▸(*b*)]. The gas was passed from the bottom to the top exhaust. The box was sealed with O-rings and electrical feedthroughs were sealed with Loctite SI5926.

**Figure 3:**
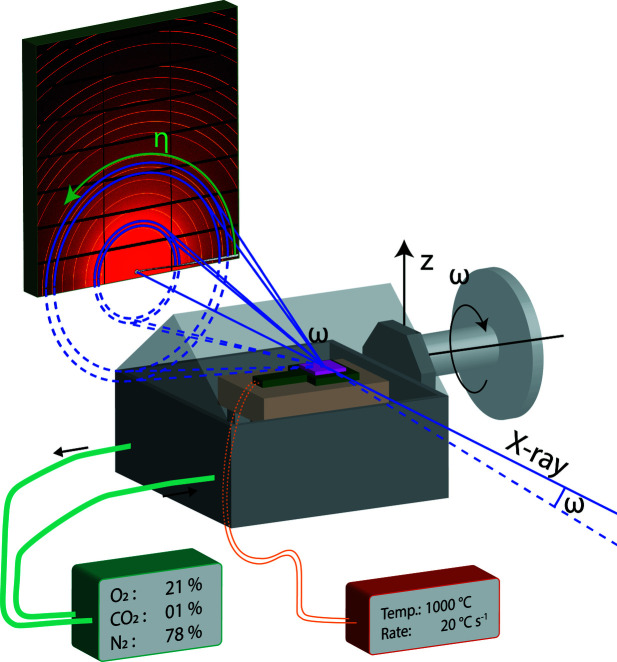
A conceptual drawing of the *in situ* thin film rapid heating setup with temperature and atmosphere control for data collection using a large 2D detector.

To have a favorable scattering geometry for thin film measurements, the near-grazing-incidence X-ray diffraction geometry shown in Fig. 3 ▸ was chosen. Performing the measurements in this geometry reduced the signal from the single-crystalline substrates, which is essential for the subsequent data treatment. The film surface was placed higher than the lower edge of the aluminium frame for the Kapton windows, making it possible to perform a sample alignment without blocking the direct beam. The beam center was placed at the bottom of the 2D detector to obtain a higher coverage of reciprocal space.

### 2.2. Thin film preparation   

All the ferroelectric thin films were prepared with the aqueous CSD method. Stable aqueous solutions of each of the metal ions were prepared and mixed in the desired stoichiometry for the given compound. The mixed solutions were spin coated on 10 mm × 10 mm single-crystalline (100) SrTiO_3_ substrates from CRYSTAL GmbH or a platinized silicon (100) [Pt/TiO_2_/SiO_2_/Si(100)] substrate from SINTEF ICT, Norway, where the surface had been plasma-cleaned in O_2_/N_2_ and dried on a hotplate at 150°C for a few minutes just after deposition in an ISO7 clean room (NTNU Nanolab). The BTO precursor solution was based on aqueous solutions of Ba(NO_3_)_2_ and titanium isopropoxide, and an aqueous solution of Ba(NO_3_)_2_, Sr(NO_3_)_2_ and Nb-complexed malic acid was used for the SBN precursor (Madaro, 2010 ▸). The precise experimental procedures for the BTO (Raeder *et al.*, 2018 ▸; Bakken *et al.*, 2020 ▸) and SBN films (Blichfeld *et al.*, 2020 ▸) are available elsewhere.

Solution concentration was around 0.25 *M* for Ba^2+^ and Ti^4+^, 0.1 *M* for (Sr^2+^ + Ba^2+^) and 0.2 *M* for Nb^5+^. These concentrations resulted in film thicknesses of 15 and ∼25 nm for annealed films of BTO and SBN, respectively.

### 2.3. Data measurement and treatment   

X-ray scattering data were recorded in near-grazing-angle geometry, after centering and alignment of the sample, using an incidence angle ω of 2° (Fig. 3 ▸). During one data set measurement, a rotation of 1° in ω was used to probe a large part of reciprocal space. One of the greatest challenges for thin film measurements under varying conditions is the change in position of the film, and this applies to the thermal expansions created in the setup during heating. To overcome this challenge, the sample was translated downwards along an axis parallel to the surface normal of the film, such that the ω axis was kept stationary in the plane of the surface of the film. The measurements were therefore performed at a set of height offsets, *e.g.* 0, −100, −200, −300 and −400 µm, that were continuously repeated. The number of offsets was determined based on the heating rate for the individual experiments. By using this measurement sequence, at least one height had the full beam hitting the sample. The beam size used was 250 µm × 500 µm for height and width, respectively. Details of the correction for using the different heights are provided in Figs. S1 to S5 in the supporting information.

The X-ray wavelength was chosen such that suppression of the fluorescence of strontium in the substrate was minimized by going below the *K*β_1_ absorption edge of 15.8357 keV = 0.782941 Å (Thompson *et al.*, 2009 ▸). The position of the detector relative to the beam and the wavelength were determined with measurements on a standard NIST SRM 660a LaB_6_ sample and the calibration module of *pyFAI* (Ashiotis *et al.*, 2015 ▸). Data integration of single detector position measurements was done with the *Bubble* software (Dyadkin *et al.*, 2016 ▸) and the post-reaction data recorded at two heights to account for the gap in the detector (inactive area between the photon counting modules) were integrated using the multi-geometry module of *pyFAI* (Ashiotis *et al.*, 2015 ▸). Data treated as single crystals were processed with *CrysAlisPro* (Rigaku Oxford Diffraction, 2013 ▸) (see Section 3.2).

## 3. Selected examples of use of the *in situ* setup   

### 3.1. Polycrystalline growth – fast heating rate   

An SBN thin film, which has a large range of application possibilities (Lukasiewicz *et al.*, 2008 ▸), was annealed with the highest heating rate (20°C s^−1^) possible for the setup. Physical vapor deposition and CSD of SBN on MgO or STO substrates have been reported because of the small lattice mismatch that could favor epitaxial growth (Infortuna *et al.*, 2006 ▸; Boulay *et al.*, 2007 ▸; Lam *et al.*, 2018 ▸; Shirokov *et al.*, 2018 ▸). Here, a single-crystalline STO (100)-oriented substrate was used with a single deposited layer of SBN with Sr:Ba = 40:60.

The substrate was oriented by visual inspection to have the *a* axis orientated parallel to the incoming X-ray beam. Four height offsets were used (−100, −200, −300, −400 µm), with the starting ω = 1°, Δω = 2° and an exposure time of 1 s. The time between two consecutive measurements was limited by the speed of the motor translating between the different heights (on average 4.5 s).

**Figure 4:**
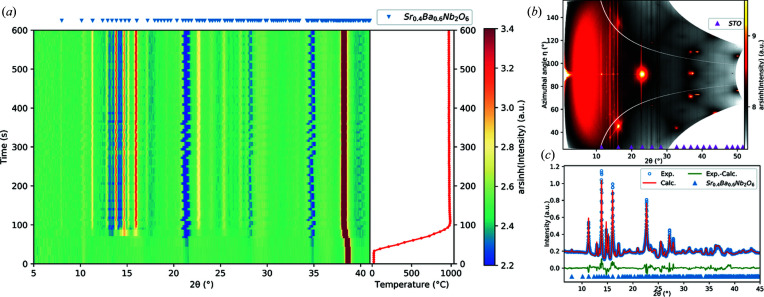
(*a*) *In situ* XRD data from the annealing of an SBN thin film on STO(100) plotted as diffracted intensity as a function of time, with the corresponding temperature in the right-hand panel. The colormap indicates the inverse hyperbolic sine (arcsinh) of the intensity to show all details in the intensity, especially with the strong (311) Bragg reflection for STO at 2θ = 38.65° at room temperature (λ = 0.780055 Å). (*b*) Summation of a room temperature ω scan covering 15° at two different detector heights, to minimize the detector gap in the data. The data show the polycrystalline nature of the thin film, where the Bragg reflections from the substrate are well confined in reciprocal space as seen from the small spread in azimuthal angle (η). A small degree of preferred orientation for SBN can be seen in the (411) peak at 2θ = 14.78° and η = 90°. (*c*) Rietveld refinement of the data set in (*b*), where the background has been subtracted. The refinement was done with March–Dollase (100) and (001) texture components and a starting model based on a Rietveld refinement of a powder data set measured on the calcined powder of the corresponding sol–gel. Details of the refinement can be found in the supporting information, Section S3.1.1.

Fig. 4 ▸(*a*) shows the time evolution of the *in situ* diffraction data, and in the right-hand panel the film surface temperature versus time is given. The heating rate, set to 20°C s^−1^, slowed down towards reaching 1000°C, giving an average heating rate of 15°C s^−1^. The crystallization of SBN initiates at 724°C. When inspecting the data set at this temperature a large degree of texture can be seen, but when the film crystallizes further the initial texture does not develop further and the rest of the sample becomes polycrystalline (see Fig. S7).

In Fig. 4 ▸(*a*), an apparent peak around 2θ = 10° is shown, and there are three regions with low intensity at 2θ = 21.3, 28.4 and 34.8°. These are all artifacts from the integration process and the gaps between the detector subsections: since a significant change in intensity along the azimuthal angle η is present, the information missing in the detector gap cannot be recreated to give a smooth background. This can be overcome if the data are measured with the detector translated to a new position without gap overlap. An example of a data set where this has been performed is presented in Fig. 4 ▸(*b*), where the data set was measured with the detector translated 10 mm upwards, in addition to the same height used for the *in situ* experiment. The data were measured at room temperature after the finished experiment, with the starting ω = −1°, Δω = 0.1° and 210 steps. Since the movement was vertical, the vertical detector gaps still overlap, giving rise to two streaks of missing information, but since these are narrow in η this does not influence the integrated data, shown in Fig. 4 ▸(*c*). The detector gaps are not static with respect to the scattering angle when the thermal shift adjustment is applied and they result in an oscillation of the positions when the datasets from individual height offsets are merged. One of the requirements for *in situ* measurements is that the time resolution is sufficiently high, therefore translation of the detector is not feasible for these types of experiment with high heating rates, since precise movement of the detector is slow.

Fig. 4 ▸(*c*) shows the integrated data from Fig. 4 ▸(*b*) where masking of the substrate diffraction lines and background subtraction have been applied, together with a Rietveld refinement of the SBN structure. Because of the preferred orientation for the initial crystallization, two March–Dollase texture components must be used to obtain a satisfactory fit. The refinement on the unfilled tetragonal tungsten bronze structure yields the cell parameters *a* = 12.4998 (11) and *c* = 3.9637 (3) Å. This fits well with the composition-dependent data given by Podlozhenov *et al.* (2006 ▸) for bulk SBN for *x* = 0.4.

Thus, an example of measuring *in situ* annealing XRD data at a high heating rate of 20°C s^−1^ and a maximum temperature of 1000°C has been demonstrated for a polycrystalline SBN thin film with a small degree of preferred orientation. The crystallization process can be investigated under the same conditions that an RTP process utilizes. The data presented in this example show that SBN crystallizes directly without the formation of any intermediate or secondary crystalline phases, which typically increase the likelihood of heterogeneous nucleation at the substrate resulting in texture. However, for this SBN film there was limited texture. Highly textured films are however possible from CSD processing, as shown in the next section.

### 3.2. Highly preferred growth of BTO   

In the pursuit of a lead-free ferroelectric material, BTO is of great technical relevance. Furthermore, the development of a CSD method for making thin films of BTO has been studied because of its low cost and relatively easy fabrication for miniature capacitors (Raeder *et al.*, 2018 ▸). The nature of the crystallization and growth is a key parameter for the functional properties of the thin film.

**Figure 5:**
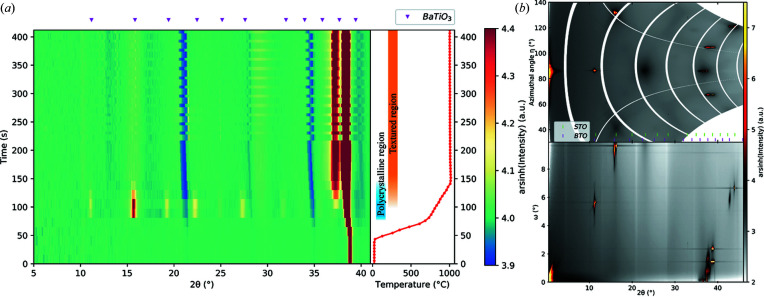
(*a*) *In situ* XRD data for a BTO thin film on an STO(100) substrate plotted as diffracted intensity as a function of time, with the corresponding temperature in the right-hand panel. The colormap indicates the inverse hyperbolic sine (arcsinh) of the intensity to show all details in the intensity. The intense peak for STO(311) is at 2θ = 38.82° (λ = 0.78242 Å) at room temperature. In the temperature versus time panel, the blue and orange bars show the time regions where BTO is polycrystalline and highly textured, respectively. (*b*) A data set measured at room temperature with the starting ω = 0°, Δω = 0.1° and 100 steps. In the top part all 100 steps have been summed, and in the bottom part each data set has been integrated without differing in η.

To investigate crystallization *in situ*, a spin-coated BTO thin film on STO(100) was utilized. Three height offsets were used (−200, −300, −400 µm), with the starting ω = 1°, Δω = 1° and an exposure time of 1 s. The heating program was designed to have three sections, first a fast heating rate of 20°C s^−1^ up to 700°C, then at 5°C s^−1^ up to 1000°C, followed by a dwell period, as shown for the actual data in Fig. 5 ▸(*a*).

Upon heating, the BTO film quickly crystallizes at ∼700°C when entering the second step in the heating program. As can be seen in Fig. 5 ▸(*a*), all Bragg peaks for BTO are visible and, together with the as-recorded frame from the detector in Fig. S9, this shows the polycrystalline nature of the sample. During the second heating step there is a gradual transformation in the intensity of the peaks, as indicated by the blue and orange bars in the right-hand panel of Fig. 5 ▸(*a*). This change follows from a change in relationship between thin film and substrate, where heterogeneous nucleation and growth of BTO are favored at high temperatures and the (311) peak for BTO is the only reflection left in the part of reciprocal space probed during the *in situ* experiment.

By performing a measurement at room temperature following the *in situ* experiment, where the sample is rotated in small steps through a larger section of ω, a larger part of reciprocal space is probed with higher resolution. This is somewhat similar to a normal rocking-curve measurement, except when using a large 2D detector much of the critical alignment is not necessary. The two panels in Fig. 5 ▸(*b*) show a data set measured with the starting ω = 0°, Δω = 0.1° and 100 steps at room temperature after the *in situ* experiment with 2θ as the *x* axis, and the data integrated for all η as a function of the ω step, and data integrated for all ω steps as a function of η, in the bottom and top panels, respectively. When combined, the data integrated along the two orthogonal axes show a high degree of texture for the BTO sample compared with the polycrystalline region during crystallization (Fig. S6).

Nucleation of polycrystalline BTO which transforms into a film with a high degree of preferred orientation indicates that the preferred orientation is a result of the growth kinetics of the film. Thus, exemplified here is the possibility of studying the crystallization of not only polycrystalline thin films but also thin films with highly preferred orientations. Having the option of obtaining the full substrate–thin film relation is of great importance: thin films for technological applications require optimized functionality, which for ferroelectricity is achieved with textured films, therefore a full understanding is key. In this BTO film the preferred orientation follows the substrate orientation, as the same reflections are present in the film and substrate, in accordance with the findings of Raeder *et al.* (2018 ▸). The crystallization behavior can be influenced by external conditions besides the intrinsic, as seen in the following section.

### 3.3. Atmosphere control during annealing   

From previous *ex situ* experiments on the crystallization of the sol used for the BTO thin film (Raeder *et al.*, 2018 ▸) and in accordance with the work of Ischenko and co-workers (Ischenko, Woltersdorf *et al.*, 2007 ▸; Ischenko, Pippel *et al.*, 2007 ▸), the existence of an intermediate oxygen-deficient calcite-type of barium carbonate, barium oxycarbonate [BaO_*x*_(CO_3_)_1−*x*_], is known. From the experiment in Section 3.2, this phase was not observed due to the fast heating rate. However, the phase was observed in the *ex situ* study of powders heated to 550°C (Raeder *et al.*, 2018 ▸), hence the inter­mediate metastable BaO_*x*_(CO_3_)_1−*x*_ phase should also form in films, under certain processing conditions. By understanding the mechanism for the formation of this unwanted phase, the effects of this phase on the final BTO film can be reduced.

The stability of carbonates is in general sensitive towards CO_2_, therefore an experiment was designed to investigate whether CO_2_ leads to stabilization of BaO_*x*_(CO_3_)_1−*x*_ and if this influences the BTO formation. By increasing the CO_2_ concentation in the atmosphere, the final step in the reaction from Ischenko, Pippel *et al.* (2007 ▸) could potentially be suppressed:
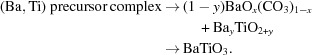



In order to control the atmosphere, an airtight lid, allowing the geometry of the setup to be preserved and not reducing the signal from the sample significantly, was designed as described in Section 2.1. For the atmosphere control, the gas handling system implemented on SNBL was used for N_2_, O_2_ and CO_2_ (van Beek *et al.*, 2011 ▸). The relative gas flow was calibrated to give the desired composition ratio of N_2_:O_2_:CO_2_ = 45:15:40. On the gas inlet to the cell two lines were fitted, the mixed gas line and a membrane pump, with valves on both lines. This allowed for performing pump–purge cycles of the desired atmosphere.

A one-layer BTO precursor film on STO(100) was measured with height offsets of −100, −200 and −300 µm, an exposure time of 2 s, the starting ω = 1° and Δω = 1° for the first 1100 s, with a heating rate of 5°C s^−1^ up to a long hold at 500°C. After this an exposure time of 10 s and measurements at −200 and −300 µm were used.

**Figure 6:**
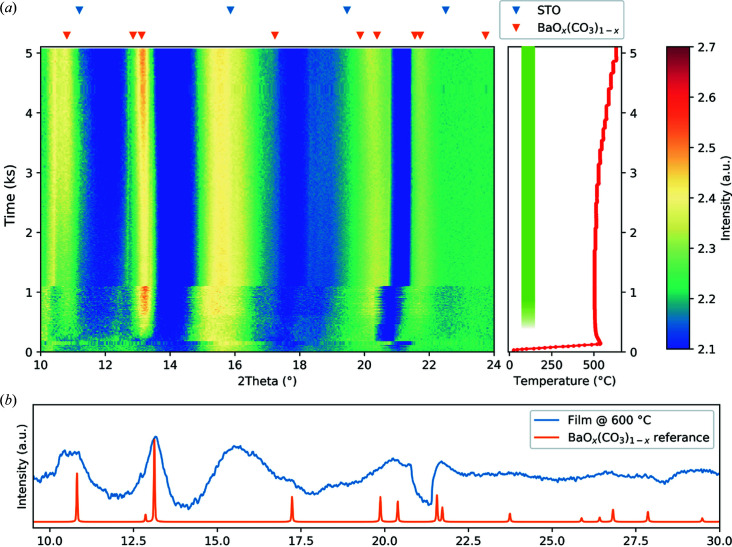
(*a*) *In situ* XRD data for a BTO precursor thin film on STO(100) in an atmosphere of concentration ratio N_2_:O_2_:CO_2_ = 45:15:40, with the corresponding temperature in the right-hand panel. The colormap indicates the inverse hyperbolic sine (arcsinh) of the intensity to show all details in the intensity (λ = 0.78242 Å). (*b*) A diffractogram of the film at 600°C compared with the BaO_*x*_(CO_3_)_1−*x*_ reference diffraction pattern. The structure of the BaO_*x*_(CO_3_)_1−*x*_ phase was obtained from Antao & Hassan (2007 ▸), ICSD 158389. The BaO_*x*_(CO_3_)_1−*x*_ phase is the only phase that formed during annealing in a CO_2_-rich atmosphere and its presence is indicated by the green bar in the temperature panel.

Fig. 6 ▸ shows the integrated data with the background subtracted to show the features present. All the regions with high intensity at the initiation of the experiment are either from the Kapton windows or the detector gaps. The signature from the sample is the two peaks at 2θ = 10.83° and 13.11° [Fig. 6 ▸(*b*)] corresponding to the two most intense diffraction lines (101) and (102) for BaO_*x*_(CO_3_)_1−*x*_ (Antao & Hassan, 2007 ▸).

The BaO_*x*_(CO_3_)_1−*x*_ phase appears in the long-hold region at 500°C and persists for the hold duration of the experiment, even when the temperature is stepwise increased by 5°C up to 630°C, and its presence is indicated by the green bar in the temperature panel of Fig. 6 ▸. This is in contrast to the data used for the example of adjustment of the detector-to-sample distance (DTSD) in Section S2, especially in Fig. S6, where a similar sample is heated without a special atmosphere at a heating rate of 5°C s^−1^ up to 700°C, where the same BaO_*x*_(CO_3_)_1−*x*_ phase is an intermediate before the crystallization of BTO.

This shows that using a CO_2_-rich atmosphere does stabilize the BaO_*x*_(CO_3_)_1−*x*_ phase at the expense of crystallization of BTO. Limiting the amount of CO_2_ in the processing atmosphere will therefore be important for obtaining phase-pure BTO films from aqueous CSD, especially at low annealing temperatures. The formation of BaO_*x*_(CO_3_)_1−*x*_ has also been reported to influence texture formation in BTO films (Bakken *et al.*, 2020 ▸), so understanding how this intermediate phase is affected by the processing parameters is of importance for fabricating high-quality BTO films.

The three selected examples showcased in this and the previous two sections demonstrate the capabilities of the presented design for a high-temperature *in situ* annealing setup for thin films to mimic the conditions found in an RTP unit that is otherwise a black box. Here all the samples were single-layered CSD ferroelectric thin films, but the setup is versatile and can be used for *in situ* studies with general XRD measurements where reflection geometry is required under applied conditions.

## 4. Conclusions   

An *in situ* synchrotron scattering sample environment for annealing/pyrolysis of thin films with a high heating rate to high temperatures with atmosphere control has been developed. The setup comprises the following components: (i) a dedicated cell proving high heating rates and atmosphere control, (ii) control electronics to operate the cell, (iii) data acquisition protocols accounting for thermal misalignment of the sample, and (iv) data processing, visualization and analysis schemes for powdered, textured and epitaxial films.

The experiments conducted under three different conditions and the resulting nature of the thin films obtained, *i.e.* polycrystalline or highly textured, demonstrate the diversity in the crystallization mechanisms in the thin films that can be characterized *in situ*. The processes investigated were firstly crystallization of an SBN thin film deposited on an STO(100) substrate with a high heating rate (20°C s^−1^) and high annealing temperature (1000°C), secondly the crystallization of a BTO thin film on an STO(100) substrate demonstrating heterogeneous growth, and finally a similar BTO thin film annealed under a CO_2_-rich atmosphere to stabilize an intermediate BaO_*x*_(CO_3_)_1−*x*_ phase in the thin film at the expense of crystallization of BTO.

Although our experiments were focused on the crystallization of ferroelectric films from CSD probed by diffraction, the above examples clearly show that the *in situ* setup can be applied to a broader class of samples and processes. The same setup can be used for pair-distribution function (PDF) measurements for structural characterization of partially disordered powder films (Dippel *et al.*, 2019 ▸; Roelsgaard *et al.*, 2019 ▸), for characterization of the orientation distribution function (ODF) of textured films, for mapping of Bragg and diffuse scattering of epitaxial films, and for specular and off-specular reflectivity measurements. Thus, the results of the present report can be viewed in a wider perspective and have several possible application areas.

## 5. Related literature   

The following references, not cited in the main body of the paper, have been cited in the supporting information: Kirby (1991 ▸); Knudsen *et al.* (2013 ▸); Taylor (1985 ▸).

## Supplementary Material

Additional details. DOI: 10.1107/S1600577520010140/co5147sup1.pdf
