# Supplementary material for: Experimental setup for high-temperature in situ studies of crystallization of thin films with atmosphere control
Source: J Synchrotron Radiat. 2020 Aug 21;27(Pt 5):1209–17. doi: 10.1107/S1600577520010140 (PMC7467347; doi:10.1107/S1600577520010140)
Supplement: Supplementary file 1 [file s-27-01209-sup1.pdf]

JOURNAL OF SYNCHROTRON RADIATION

Volume 27 (2020)

Supporting information for article:

Experimental setup for high-temperature *in situ* studies of crystallization of thin films with atmosphere control

Anders Bank Blichfeld, Kristine Bakken, Dmitry Chernyshov, Julia Glaum, Tor Grande and Mari-Ann Einarsrud

S1. Details of the experimental setup

In Figure S1 the details of the inner parts of the setup is shown. Figure S1(a, b) shows the design of the heating plate part. A ceramic block was shaped to fit the heating plate and a hole for the control thermocouple that is embedded halfway (Raeder *et al.*, 2018)through the heating plate. The ceramic block is held in place by four threaded steel rods, and again attached to a stainless steel “x”, which is resting on an aluminum block. The wires of the heating element are protected by an additional ceramic block on top, and covered by insulating tubing below the ceramic block, where the temperature is less than 150 °C. The ends of the wires are connected to high temperature cables through a ceramic terminal block (not shown). Figure S1(c) shows all parts required for running an experiment, except wiring. Two K-type thermocouples were used; where one was placed inside the heater and another one mounted on the surface for measuring the temperature and acting as a spring to ensure good thermal contact.

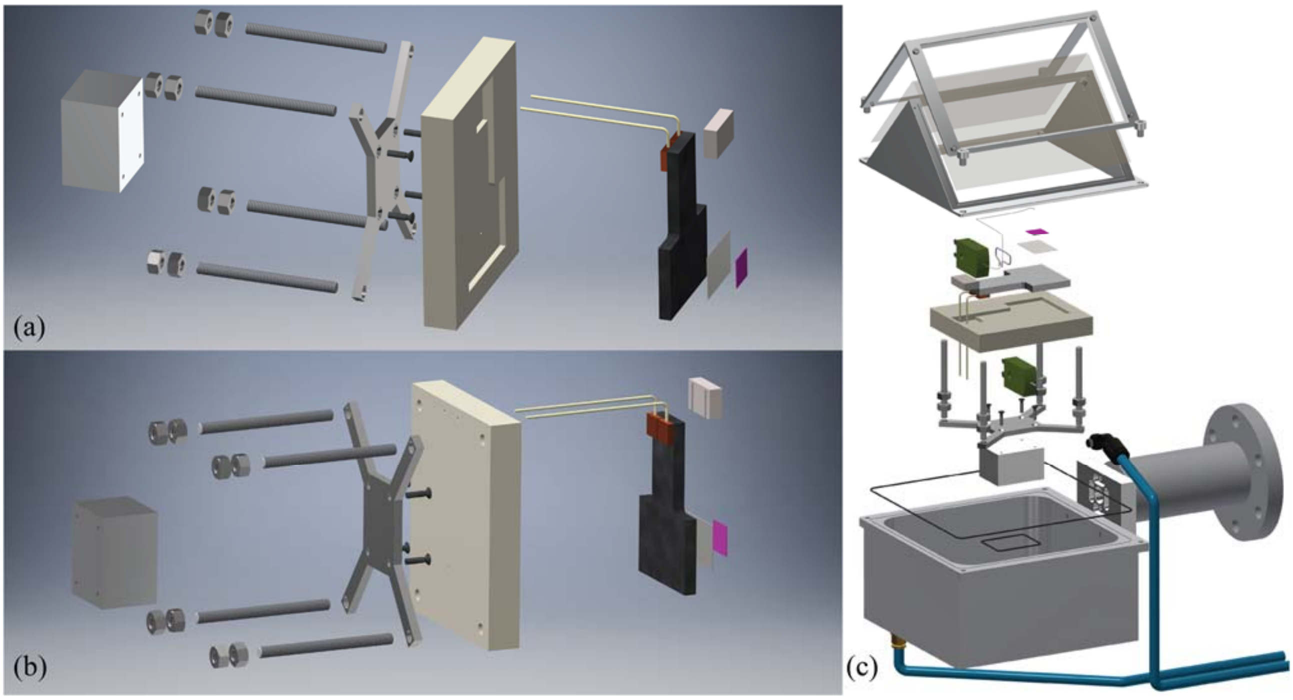

Figure S1 Explosion of the CAD rendering of the inner, (a) and (b), and outer (c) parts of the heater setup.

S2. Data treatment for cycling of height-offset

In order to handle the effective offset in detector-to-sample distance (DTSD) a Python script was developed using the modules FabIO (Knudsen *et al.*, 2013) and pyFAI (Ashiotis *et al.*, 2015) for evaluating when a given dataset was suitable for further refinement. A dataset would not be applicable if, the beam does not hit the sample or if the footprint of the beam on the sample is too small for the

data to represent an average the sample. The evaluation was based on a combination of thermal expansion of an internal standard, *i.e.* the substrate peaks for STO or Pt for platinized Si, and knowledge of linearity in the offset in the height of the sample. The thermal expansion data for STO and Pt were obtained from (Taylor, 1985) and (Kirby, 1991), respectively.

As an example, the procedure for adjustment of height offset effect on the DTSD will be described for a Pt/TiO_2/SiO_2/(100)Si substrate and a one-layer amorphous BTO film. The lattice parameter of Pt was refined for the aligned sample at RT with a LeBail fit. With the obtained cell-parameter a calibrant file for pyFAI was created, and the DTSD was refined with a fixed wavelength for the three other heights available at RT, an offset of 100, 200, and 300 µm downwards compared to the optimized height found from alignment of the sample. At an offset of 400 µm the sample was completely out of the beam. The DTSD for the RT offsets can be seen in Figure S2 as the blue series. Since the sample is translated downwards, the effective DTSD is decreased as the data were measured since the sample was tilted $\omega = 2°$ initially and rotated $\Delta\omega = 1°$ during the measurement, as illustrated in the inset of Figure S2. When the sample was shifted downwards so much that the beam shoots above the sample, the effective footprint becomes smaller, and the change in DTSD deviates from being linear, and approaches a constant as the bottom of the beam reaches the upper edge of the sample. As long the refined DTSD follows the linear trend the dataset be used for further data treatment.

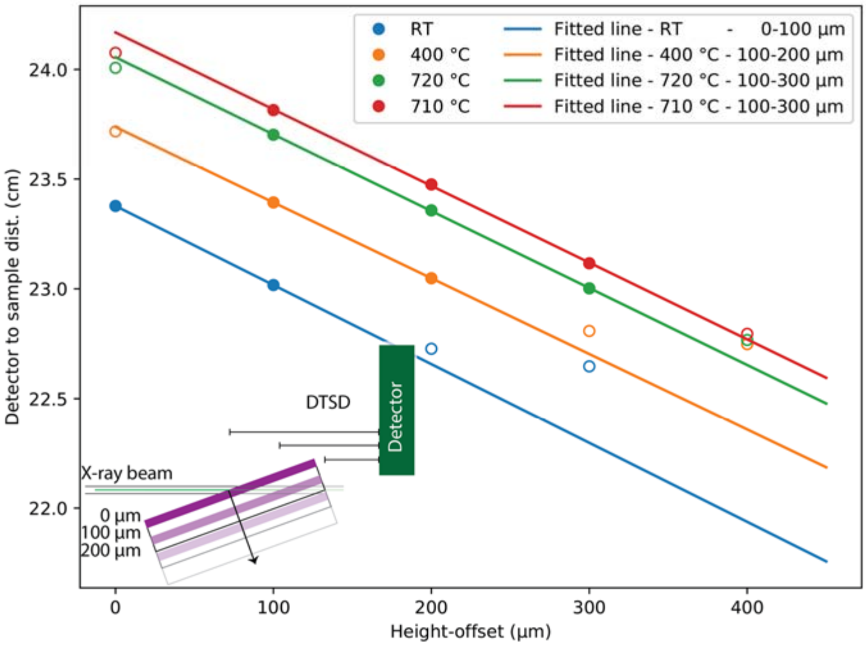

Figure S2 Refined detector-to-sample distance (DTSD) as function of height-offsets at different temperatures for a BTO sample on platinized Si. The straight lines are fits to the full colored data

points, also indicated at the end of the label in the legend, *e.g.* the fitted line for 710 °C is only fitted for the 100-300 µm offset. For the datapoint at 400 µm for RT, the sample was completely out of the beam, hence no offset could be determined. The illustration at the bottom shows, how the DTSD decreases as the sample is translated downwards. The green line in the illustration represents the center of the X-ray beam.

The data in Figure S2 were measured as part of an *in situ* experiment with a heating ramp of 2 K s^{-1} and the temperatures are shown in Figure S3. By deciding which height offsets that still have the full footprint on the sample, more data points can be used, thereby giving more information than by just using one set of height offsets as the temperature increases.

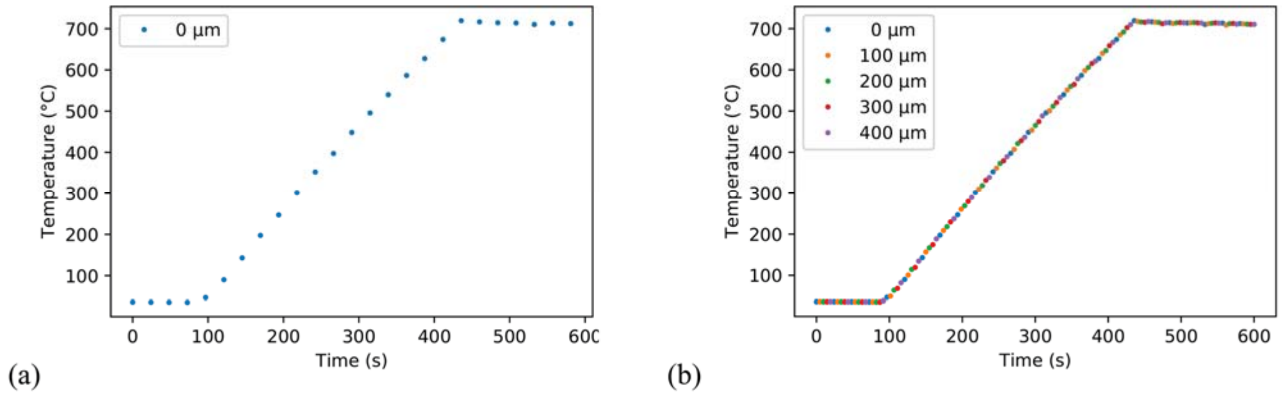

Figure S3 Plots of the sample surface temperature when the dataset where measured as a function of time for the original height, (a) and all heights (b).

Using the thermal expansion coefficient for Pt, individual calibration files where generated for a set of height-offsets at ~ 400, 720 (when the constant temperature is reached), and 710 °C (at the end for the constant temperature), and the DTSDs were manually refined for all datasets, and shown in Figure S2, with the orange, green, and red data series, respectively.

A region in 2θ for the full dataset series integrated with a DTSD set to the value at RT and 0 µm height offset, can be seen in Figure S4(a). The resulting effect of the change in DTSD can easily be seen from the five data series; the position of the peaks in the first dataset in each series should align at the same 2θ-value if the DTSD was correct. The position of the (111) and (200) peaks for Pt at RT are indicated with red dashed lines. In Figure S4(b), the dataset series with corrected DTSD according to the linear trend in Figure S2 at RT can be seen. There is a clear change in the peak positions as the sample is heated, where the positions of the peaks move to higher 2θ, which could be mis-interpreted as a unit-cell contraction. This effect arises from the thermal shift of the sample environment, the effect which was anticipated, and is the reason for performing the height-offset in the first place.

In Figure S2, the DTSD for the 100 µm offset series falls on the linear trendlines for the whole temperature range and can therefore be used as a starting point for correcting for the thermal shift of

the setup. The green lines in Figure S4, indicate the maximum for the Bragg peaks expected as a result of the thermal expansion for Pt. To correct for the thermal shift, a pseudo-Voigt function was fitted to the data series for the (111) peak, to find the difference between the expected and the observed position. Since the data have been integrated using the DTSD for the first RT dataset, and are therefore in the 2θ-space, an additional correction to the shift needs to be accounted for because the data are collected on a flat detector, resulting in a nonlinear shift in 2θ-space. The situation is shown in Figure S5, where $\tan(2\theta) = \frac{b}{L_2}$, $\tan(\tau) = \frac{b}{L_1}$, where L_1 is the DTSD that was used for integrating the dataset before the height correction, L_2 is the new DTSD that is needed to integrate the data correctly, 2θ is the calculated peak position at the temperature the dataset is measured at and corrected for thermal expansion, and τ is the peak position for the specific Bragg reflection integrated with L_1. By isolating the correct DTSD, L_2:

$$\tan(2\theta)\, L_2 = \tan(\tau)\, L_1 \Leftrightarrow$$

$$\frac{\mathrm{L}_2 = \mathrm{L}_1 \tan(\tau)}{\tan(2\theta)},$$

the data can be reintegrated and used for further data treatment. This formulation was used for the 100 μm data series, and the results are shown in Figure S4(c).

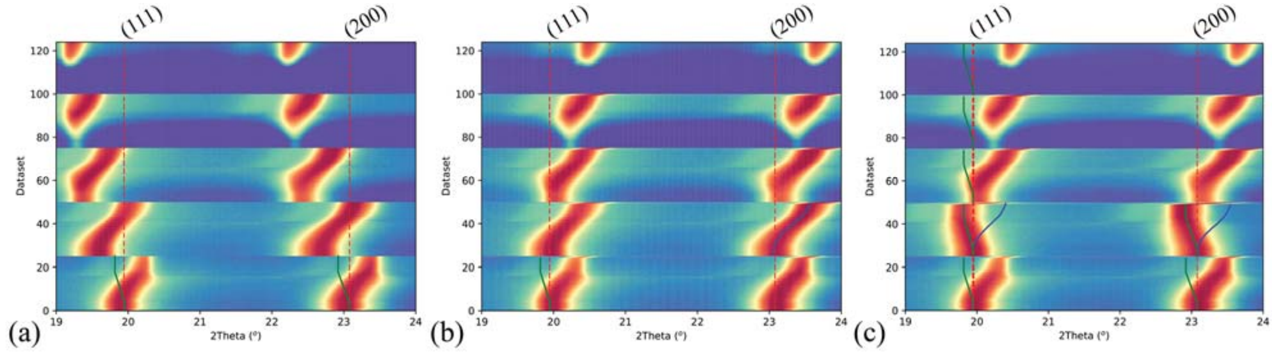

Figure S4 Selection of the 2θ range showing the (111) and (200) peaks for Pt for all datasets, indicated with red dashed lines. The datasets correspond to a height-offset of 0, 100, 200, 300, and 400 μm for datasets 0-24, 25-49, 50-74, 75-99, and 100-124, respectively. The temperatures increase from the bottom for each dataset and are shown in Figure S3. The blue lines correspond to the refined maximum of the (200) peak for the 100 μm series to a pseudo-Voigt function. The green lines show the expected peak position if there would be no thermal shift of the setup. (a) shows the all datasets integrated with a DTSD that is correct for the RT data for the original height, 0 μm. (b) The datasets adjusted to follow the linear change seen in Figure S2 in DTSD at RT. In (c) the datasets with an offset of 100 μm has been corrected to follow the expected peak position due to thermal expansion of the Pt, the others are the same as (b).

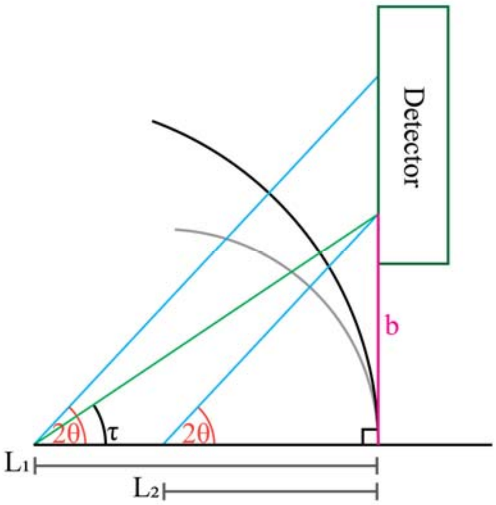

Figure S5 Sketch for deriving the relationship between the shift for a Bragg peak in 2θ-space and the correct DTSD, L_2.

The linear translation from Figure S2 can be applied to the DTSD for the 100 μm offset, corrected for thermal shift and expansion, to obtain the correction for all other heights. Here, the average of the slope of the dataset collected at elevated temperatures was -3.48(2) cm mm^{-1}. By fitting the position of the STO-(111) peak for the corrected data, the datasets that have a too large difference, can be excluded because they do not have the full footprint of the beam on the sample. In Figure S6(a), all the datasets corrected in this fashion are shown. Comparing Figure S6(b) to Figure S3, shows the data sampling interval and the regions where datasets are used. Figure S6(c) shows the final merged corrected datasets, which can be further processed as a regular time-resolved dataset from synchrotron *in situ* measurements.

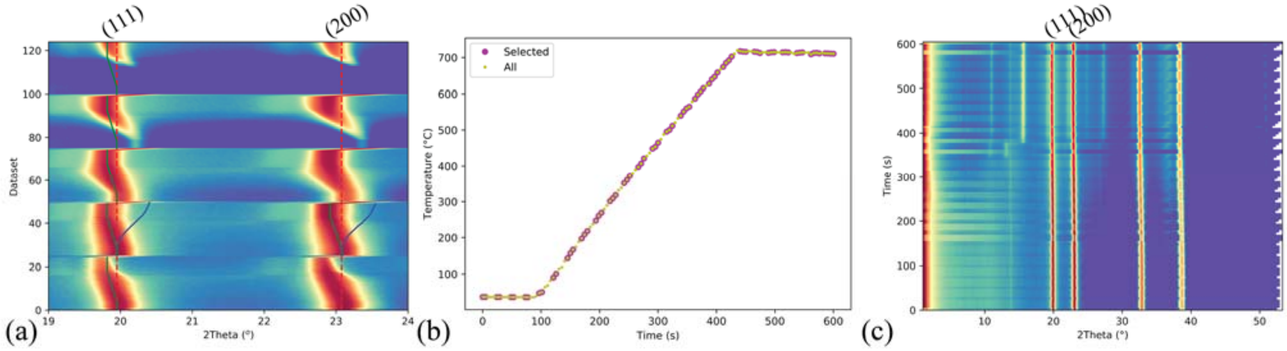

Figure S6 (a) All dataset series integrated with adjusted DTSD in comparison to Figure S4. (b) Sample temperatures for all datasets compared to selected datasets, based on the criterion for a dataset to be suited for further analysis. (c) Colormap plot of the merged dataset based on the selection in (b). The (111) and (200) peaks for the STO substrate are indicated for comparison with Figure S4.

The adjustment in DTSD is effectively a remapping of the data bins for the scattering angle, which can easily be seen at the edge for the high-2θ region in Figure S6(c) as the fluctuations in the highest

value for the datasets as function of time. At the same time, the scattering from the air in the low 2θ-region varies for each height offset, giving rise to a non-continuous change in the background level as a function of time, which is accounted for in the subsequent data analysis.

For samples where the substrate is a standard single crystal, the availability of a Bragg reflection can be limited. The strategy for data measurements must account for this, since a reference peak is necessary for adjusting the DTSD. For the STO(100) substrate the {311} peaks fits well with an ω = 2-3° and the X-ray beam approximately parallel to the {100} side of the substrate. Since the Bragg reflections for single crystals, and especially large crystals, are a convolution of multiple components the resulting shape when the data are integrated can be complex. For the samples in this study, the position was found based on the smoothened first derivative of the data in a small region around the {311} peak in 2θ to find the edges of the peak and an average of those for the position.

The precision of the data after adjusting for the height offset, is estimated to be on the fourth digit, but significant differences in the peak profile for peaks for the thin film, can occur and could lead to a lower precision.

S3. Support for the experiments

S3.1. SBN

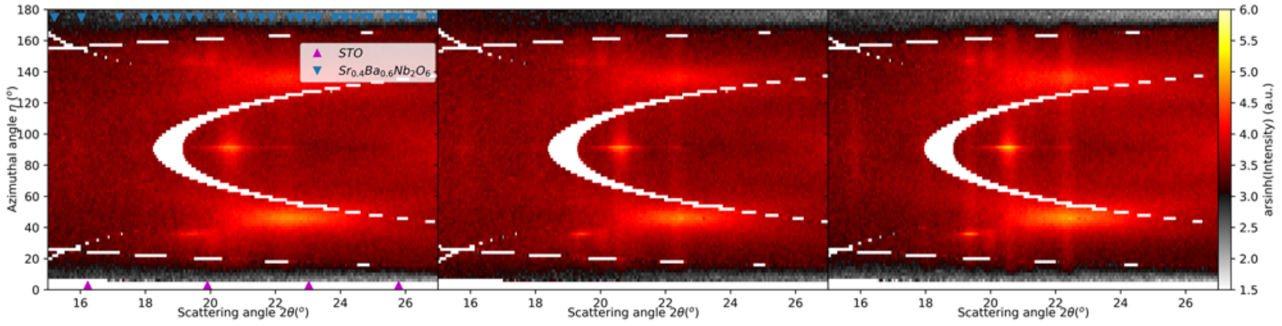

Figure S7 Data at three different temperatures that show the crystallization behavior of SBN, at 724, 784, and 916 °C, for the left, middle, and right panel, respectively.

S3.1.1. Rietveld refinement

For the Rietveld refinement of the unfilled tetragonal tungsten bronze structure of SBN, the peak profile was refined using the Caglioti pseudo Voigt function with three and one terms for the Gaussian and Lorentzian contributions, respectively. The scale factor and zero-point offset were refined. For the structure, the unit cell parameters were refined along with texture as a two term March-Dollase model for the {100} and {001} families. The structural model for SBN is given in Table S1. The refined cell parameters where 12.4998(11) and 3.9637(3) Å for *a* and *c*, respectively. The texture yielded 6.79 and 0.75 for the {100} and {001} families, revealing a small degree of preferred orientation. An R_{wp} = 1.28 and a Goodness of fit (χ^2) = 0.44 were obtained. The obtained

The low Goodness of fit is due to addition of the texture modeling adding several additional parameters (five more) to the refinement and the starting model based on a Rietveld refinement on a powder dataset measured on the calcined powder of the corresponding sol-gel. An χ^2 = 1.03 and R_{wp} = 2.95 is obtain for a refinement with a polycrystalline texture model. The visual inspection of data (Figure S8) compared to the refinement shown in **Error! Reference source not found.**(c), shows that the texture model is needed but do not hold any statistical significant information.

Table S1 Structural parameters used for the SBN phase in the Rietveld refinement with space group $P4bm$.

Label	Atom type	Occupancy	x	y	z	B_{iso} [Å^2]
**A1**	Ba^{2+}	0.8354	0.17214	0.67214	0.49324	0.02
**A1**	Sr^{2+}	0.0823	0.17214	0.67214	0.49324	0.02
**A2**	Sr^{2+}	0.6652	0	0	0.4892	0.02
**B1**	Nb^{5+}	1	0.5	0	0.01333	0.02
**B2**	Nb^{5+}	1	0.073922	0.211046	0.00295	0.02
**O1**	O^{2-}	1	0.28338	0.78338	0.9708	0.02
**O2**	O^{2-}	1	0.13965	0.07009	0.9572	0.02
**O3**	O^{2-}	1	0.99341	0.3438	0.9605	0.02
**O4**	O^{2-}	1	0.5	0	0.4752	0.02
**O5**	O^{2-}	1	0.0762	0.20507	0.4651	0.02

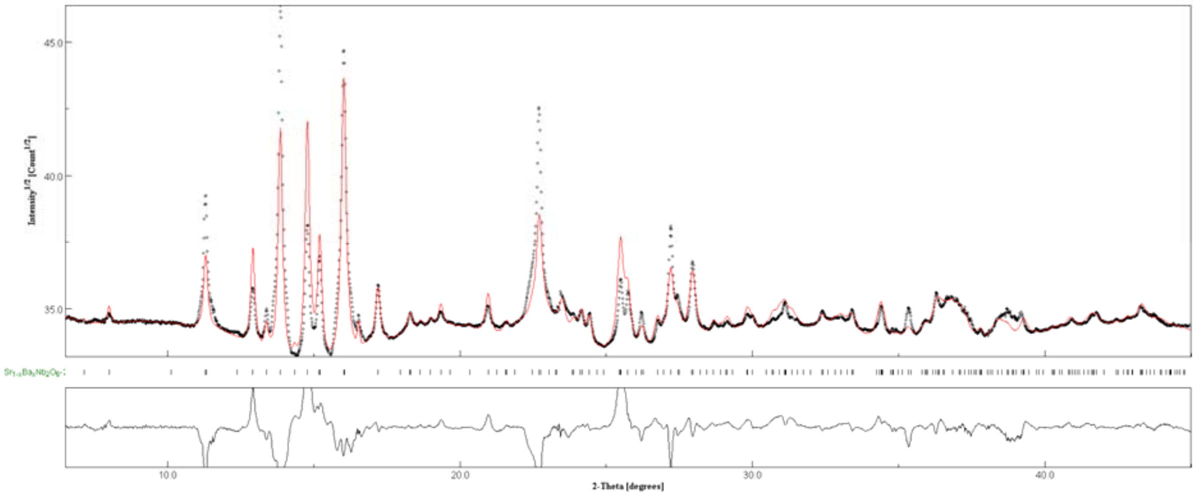

Figure S8 Rietveld refinement with out texture for SBN. To be compared with **Error! Reference source not found.**(c).

S3.2. BTO

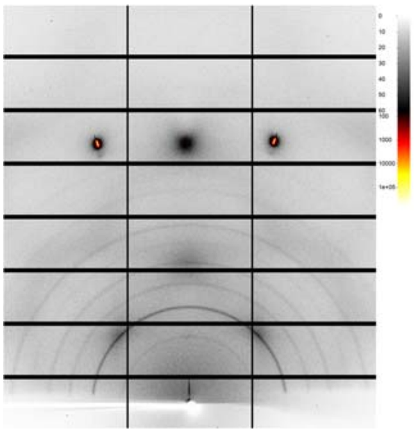

Figure S9 As-recorded detector frame from second heating step during *in situ* crystallization of BTO at 733 °C, showing the polycrystalline nature of the thin film and the {311} peaks from the STO(100) substrate.

References

Ashiotis, G., Deschildre, A., Nawaz, Z., Wright, J. P., Karkoulis, D., Picca, F. E. & Kieffer, J. (2015). *J. Appl. Crystallogr.* **48**, 510-519.

Kirby, R. K. (1991). *Int. J. Thermophys.* **12**, 679-685.

Knudsen, E. B., Sorensen, H. O., Wright, J. P., Goret, G. & Kieffer, J. (2013). *J. Appl. Crystallogr.* **46**, 537-539.

Raeder, T. M., Bakken, K., Glaum, J., Einarsrud, M. A. & Grande, T. (2018). *AIP Adv.* **8**, 105228.

Taylor, D. (1985). *Trans. J. Br. Ceram. Soc.* **84**, 181-188.
